# Psychometric Evaluation of the Postoperative Recovery Profile

**DOI:** 10.1155/2023/3745570

**Published:** 2023-05-29

**Authors:** Jenny Jakobsson

**Affiliations:** Faculty of Health and Society, Department of Care Science, Malmö University, Malmö, Sweden

## Abstract

**Aim:**

To further evaluate the postoperative recovery profile regarding its psychometric properties.

**Background:**

The postoperative recovery profile is an instrument for the self-assessment of general postoperative recovery that has received increased attention within nursing research. However, psychometric evaluation during development was sparse.

**Design:**

Psychometric evaluation was done using classical test theory.

**Method:**

Data quality, targeting, reliability, and scaling assumptions were measured. In addition, confirmatory factor analysis was used to evaluate construct validity. Data collection was made during 2011–2013.

**Result:**

Data derived from this study showed acceptable quality; however, item distribution was skewed, with ceiling effects in the majority of items. Cronbach's alpha showed high internal consistency. Item-total correlations indicated unidimensionality, whereas six items demonstrated high correlations pointing at redundancy. The confirmatory factor analysis confirmed problems related to dimensionality as the five proposed dimensions were highly correlated with each other. Furthermore, items were largely uncorrelated with the designated dimensions.

**Conclusion:**

This study shows that the postoperative recovery profile needs to be further developed to serve as a robust instrument within nursing as well as medical research. Arguably, values from the instrument should not be calculated at a dimensional level for the time being because of discriminant validity issues.

## 1. Introduction

Recovery after a surgical intervention has different meanings depending on whom it concerns. For the anesthesiologist, recovery means the return of vital reflexes when awakening from anesthesia [1, 2]. For a surgeon, short-term recovery equals home-readiness, while long-term recovery is conditioned by normal functioning and the resumption of normal, daily activities [3]. Consequently, there are several instruments measuring postoperative recovery from different points of view. However, patient-reported outcomes (PRO.s) are of utmost importance when measuring postoperative recovery; consequently, several instruments have been developed with that purpose during the past decades.

Patient-reported outcomes are measures that concern patients' health, quality of life, or functional status associated with healthcare or treatment and are reported by patients themselves [4]; (p.62). Acknowledging PROs can contribute to the delivery of high-qualitative, patient-centred care, and for this purpose, the need for patient-reported outcome measures (PROM) have increased. Using PROMs will provide a better understanding about the impact surgery has on patients' lives [5]. This, together with medical improvements, can advance the field of surgical care. Within the sphere of surgical research, there are numerous PROMs frequently used, for example, the Medical Outcome Study 36-Item Short-Form Health Survey (SF-36) [6]; the EuroQoL 5-Dimensions (EQ-5D) [7]; and the European Organization for Research and Treatment of Cancer Quality of Life Questionnaire (EORTC-QLQ) [8]. However, these are generic measures of health and should not be used if postoperative recovery is the specific outcome of interest. Instead, instruments that are developed with that specific purpose needs to be used and, most importantly, such instruments should be valid and reliable. Despite this, the majority of instruments are claimed to fail in robustness, leading to uncertainties when using the information provided [9]. In a systematic review aiming at evaluating psychometric properties of PROMs used in research for measuring recovery after abdominal surgery, Fiore et al. [10] found 22 different PROMs. In the review, 74% of the PROMs received only poor or fair quality ratings. Most frequently appraised were the three versions of quality of recovery (QOR-9, QOR-15, and QOR-40) and the abdominal surgery impact scale (ASIS); however, both instruments showed significant limitations regarding psychometric properties, except for the ASIS, which showed a high content validity. The use of PROMs for the evaluation of postoperative recovery is highly relevant. However, using instruments that are developed for a different reason or are poorly functioning might be counterproductive and result in low-quality care and reduced opportunities for enhancing patients' recovery. Most importantly, it will not benefit the patients.

One PROM that has increased in use within nursing research during recent years is the postoperative recovery profile (PRP) by Allvin et al. [11]. The instrument has been used for patients recovering from general and orthopedic surgery [12–15], heart and lung transplantation [16, 17], upper abdominal cancer surgery [18], gastric bypass [19], colorectal cancer surgery [20–22], trauma [23], and coronary artery bypass grafting [24]. The PRP was developed in Sweden, but the instrument has also been translated and used in the United States [25]. In the systematic review by Fiore et al. [10], the PRP received high ratings regarding content validity, but it could not be judged regarding another psychometric aspect since information in published studies from the development was missing.

The PRP was developed based on a concept analysis that provided a definition of postoperative recovery [26] as well as from focus group interviews with patients, nurses, and surgeons describing their understanding of postoperative recovery [27]. From the formulated definition and subsequent interviews, the developers selected 19 items and divided them into five dimensions. The PRP was assigned features to serve as a multi-item, multidimensional instrument for the self-assessment of general recovery after surgery. The PRP has been evaluated regarding content and face validity, reliability, and construct validity. The initial evaluation resulted in a minor revision of the layout, and the instrument showed high test-retest reliability [11]. Construct validity was tested on the final version pointing at a good construct validity [28]. However, later studies using the PRP have expressed concerns, especially about the scoring procedure when analyzing and interpreting results from the PRP, suggesting that the scoring might be insensitive, both at the item and dimensional levels [20, 21]. Further potentially problematic issues arise from reviewing the developmental process in more detail. First, despite some reported validity and reliability issues for two of the 19 initial items (*appetite changes* and *interest in surroundings*), all items were included in the final version of the instrument [11]. Second, it is unclear how the items were divided into five dimensions as that process is not described. Furthermore, the objective behind the scoring procedure at a dimensional level is not explained or justified.

The increasing use of the PRP within nursing research demonstrates a continuing need for an instrument that adequately measures PRO after surgery. However, to ensure the trustworthiness of studies building their results on the PRP, the instrument needs additional evaluation.

## 2. Methods

### 2.1. Aim

This study aims to further evaluate the postoperative recovery profile regarding its psychometric properties.

### 2.2. Design

A psychometric evaluation of a 19-item questionnaire intended to measure postoperative recovery.

### 2.3. Participants

This study utilizes data from a larger data collection prospectively following the recovery process in patients after colorectal cancer surgery. A consecutive recruitment was made at a university hospital in Sweden. Eligible patients had a cancer in the colon or rectum and were planned to undergo surgery to remove the tumor. Hence, participants were recruited at their preoperative informational visit before surgery. The inclusion criterion was the ability to understand and respond to the instrument in Swedish.

### 2.4. Data Collection Procedure

Patients who agreed to participate received the PRP instrument one month after surgery. The PRP was distributed by regular mail together with a prepaid envelope for return. Two reminders were sent to those who did not return the instrument.

### 2.5. Instrument

The PRP consists of 19 items that represent symptoms that can arise during the postoperative recovery process, for example, *pain*, *nausea*, or *problem with emptying the urinary bladder*. The items are formulated as statements, and patients are asked to indicate how much they experience each symptom, for example, “right now I feel a pain that is….” The response alternatives are “none,” “mild,” “moderate,” and “severe.” The recommended scoring at the item level is made by counting all the items responded to by “none.” The number of “none” responses constitutes an indicator sum and equals the level of recovery (Table 1). In order to assess recovery at a dimensional level, the developers have described that level of recovery in each dimension should be based on the most severe problem reported by the patient [29]. For example, the dimension *physical function* includes five items. According to the proposed scoring procedure, level of recovery should be assessed as “severe” if the patient reports “none,” “none,” “severe,” “mild,” and “mild” since the most severe problem direct the scoring.

The instrument has a second version with 17 items instead of 19. It excludes the items concerning *sexual activity* and *reestablishing everyday life* and is intended to measure recovery while patients are hospitalized. For this current study, the version with 19 items was used since included patients responded to the instrument after discharge from hospital.

### 2.6. Data Analysis

According to the original recommended scoring procedure of the PRP at an item level, as described above, recovery should be evaluated by calculating an indicator sum based on items responded by “none.” However, there have been concerns about this scoring procedure because it excludes all possible answers except the “none” answers. To better reflect the full range of recovery, the scoring procedure at the item level was revised in this current study to include all response alternatives. Hence, a total score was calculated according to the following: “severe” = 1, “moderate” = 2, “mild” = 3, and “none” = 4. Thus, the total score could range between 19 and 76. A higher total score means better recovery. Current study did not propose a revised scoring procedure at the dimensional level.

The data were initially analyzed using classical test theory (CTT) to explore data quality, targeting, reliability, and scaling assumptions. In addition, a confirmatory factor analysis (CFA) was performed to evaluate construct validity. Classical psychometric tests were made using the IBM SPSS Statistics (version 28) and the CFA using IBM SPSS Amos (version 28).

### 2.7. Data Quality

A high proportion of missing data leads to uncertain results. Therefore, data quality was examined regarding missing data for items and computable scale scores. The proportion of missing data for items should be less than 10%, and in case of missing items, the scale score was considered as computable if more than 50% of the items were completed [30].

### 2.8. Targeting

In order to evaluate whether the PRP instrument targeted the full variance within the sample, floor and ceiling effects as well as skewness were calculated. Floor and ceiling effects were considered as present if the proportion of answered response alternatives exceeded 20%. Furthermore, the skewness range should be between −1 and 1 [30, 31].

### 2.9. Reliability

Because this study is based on previously collected data, no test-retest reliability was measured. Therefore, reliability was measured only regarding internal consistency using Cronbach's alpha. Alpha coefficients >0.8 were considered as acceptable [30, 32].

### 2.10. Scaling Assumptions

In a unidimensional scale, all items contribute equally to the total score. Furthermore, Likert-based items can be legitimately summed if they have approximately the same mean values and standard deviations (SD) [30]. To evaluate this, item response distributions were reviewed. In addition, item-total correlations were calculated. The correlation values were considered as satisfactory when ranging between 0.40 and 0.70 [31].

### 2.11. Construct Validity

To examine construct validity of the PRP dimensions and how well items represented the dimensions, a CFA was performed. Cases with missing items were excluded from the CFA; thus including 122 cases.

To indicate how well the five dimensions, proposed by Allvin et al. [28], fitted the sample data, the model fit was assessed using relative/normed chi-square statistics (CMIN/DF), goodness-of-fit statistics (GFI), adjusted goodness-of-fit statistics (AGFI), the standardized root mean square residual (SRMR), and the root mean square error of approximation (RMSEA). Furthermore, a comparative fit index (CFI) was used to test the hypothesis that all dimensions in the model were uncorrelated, thus pointing at good discriminant validity [33, 34].  Table 2 present the thresholds for model fit. In addition, correlations between dimensions as well as item loading scores were examined. As the PRP is supposed to be multidimensional, assessing different aspects of postoperative recovery, its dimensions were expected to be uncorrelated with each other. In contrary, the items within each dimension were expected to show high correlations with their respective dimension. Correlations between dimensions should therefore not exceed 0.80, and item loadings should be above 0.70.

### 2.12. Ethical Considerations

The study was approved by the Swedish Ethical Review Authority before the study started (No. 2011/451; 2021-06818-02). It was also conducted in line with the ethical principles expressed in the Declaration of Helsinki [35]. All eligible patients were approached with verbal and written information containing the aim of the study, a description of the study procedure, an assurance of confidentiality, and the right to withdraw at any time. Patients who did not return the instrument despite two reminders were considered as having withdrawn and did not receive further reminders.

## 3. Results

In all, 154 patients participated in the study. Those were equally distributed based on gender with a mean age of 69.4 years (SD 10.9). The majority of patients had undergone a low anterior resection of the rectum. Further participant characteristics are displayed in Table 3. Results from the psychometric evaluation are presented below.

### 3.1. Data Quality

The percentage of missing data for items was acceptable varying from 0 to 1.9%, except for the item *sexual activity*, which had 15.6% missing data (Table 4). The proportion computable scale score was 79.2%.

### 3.2. Targeting

Regarding the total score, there were no floor and ceiling effects present (0.6% and 3.9%, respectively). However, the item distribution was skewed (−1.167) with a total mean score (62.27) close to the maximum value. Reviewing each item revealed considerably high proportion of ceiling effects in all items except one (ranging from 17.8% to 84.9%), pointing to the instrument being unspecific (Table 4).

### 3.3. Reliability

Cronbach's alpha was 0.927, indicating high internal consistency in the sample. If the item *gastrointestinal function* was deleted, the alpha would only be slightly improved (0.929).

### 3.4. Scaling Assumptions

In general, mean scores were high (ranging from 2.78 to 3.78), but item response distributions showed relatively equivalent mean scores and SDs, justifying the items to be summed. One item, *sexual activity*, presented an SD of 1,258, which was considerably higher than those for other items. Item-total correlations exceeded 0.40 for all items except one with a slightly lower, but still acceptable, correlation (0.355). This indicates that the instrument might be unidimensional. Moreover, there were six items with a correlation >0.70, indicating a potential redundancy (Table 4).

### 3.5. Construct Validity

In the CFA, model fit showed a generally acceptable fit to sample data (Table 2). The GFI and AGFI were lower than the desirable amount. The CFI was close to acceptable.

**Figure 1:**
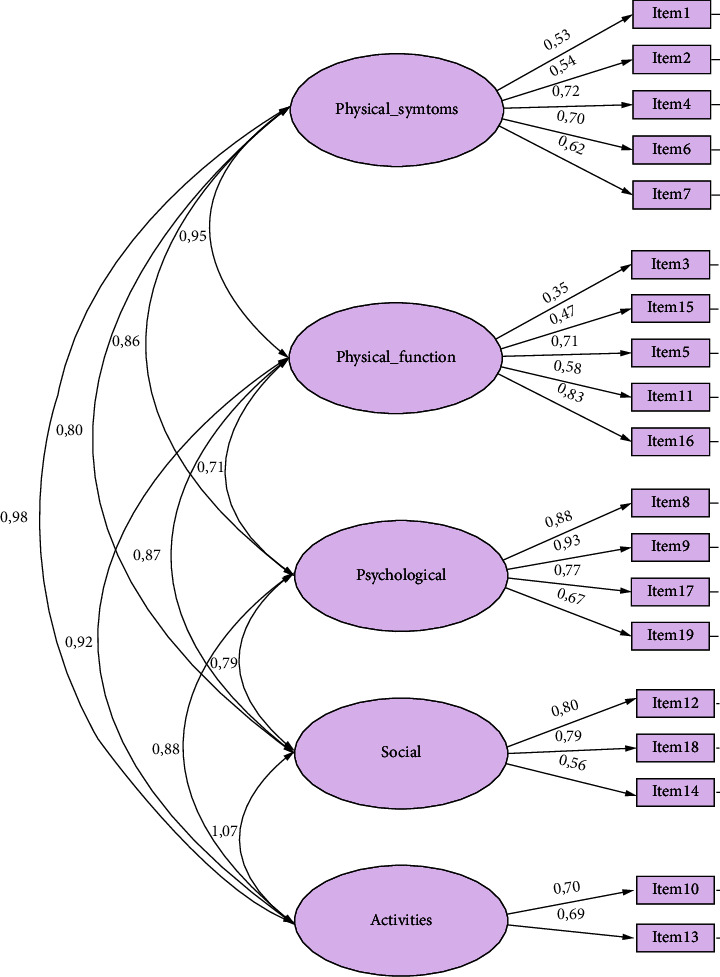
Path diagram from CFA showing correlations between dimensions and between dimensions and respective items.

As displayed in Figure 1, high correlations between dimensions were shown, except between the dimension physical function and dimension psychological function (0.71) and between the dimension psychological function and dimension social function (0.79). Furthermore, most items showed low correlations with their respective dimensions. The highest correlations were seen for item 8 (*anxiety and worry*) and item 9 (*feeling down)* and the dimension *psychological function* (0.88; 0.93), proving them useful for measuring the psychological aspects of recovery. Moreover, item 12 (*social activities)* showed acceptable correlation with the designated dimension *social function* (0.80).

## 4. Discussion

The study by Fiore et al. [10] indicates that there is an apparent risk that weak instruments are being applied in research and for clinical decision making. When the PRP was developed, it initially demonstrated promising results regarding its psychometric properties, but it was never fully evaluated. The results from this study clearly show that the PRP has potential but needs to be further developed.

A good instrument should have the ability to target the full variance within the sample. If not, valuable information is lost. Therefore, the previously suggested scoring procedure, namely counting the “none” responses to produce an indicator sum, is problematic as it only considers one response option. Hence, targeting becomes limited. In addition, by using such a scoring procedure, it could be argued that one does not measure postoperative recovery. Instead, it measures patients that are more or less fully recovered, and doing so is of minor scientific and clinical value. Scientifically, there is a need to discover the normal pattern of recovery, and clinically, there is a need to identify patients who do not follow the expected, normal pattern. In this study, the total score was calculated, and the results showed that the data had a positive skewed item distribution. Although there were no ceiling effects regarding the total score, the total mean was close to the maximum score. At an item level, there was a considerable high proportion of ceiling effects for almost all items. This is another argument for not using the previously proposed scoring procedure, only accounting for the “none” options, but beyond that, the results suggest that the scaling does not satisfy criteria for acceptability [30]. Despite changing the scoring procedure, there are still problems with covering the entire range of the scale. It might be that the response options are too few, although it has been discussed whether an increase in the number of response options will enhance validity if the response options are unable to distinguish differences [36]. If going forward with the development of the PRP, it would be beneficial to test an expansion of the response alternatives to include at least five options as well as check that the wording of the items functions as intended. Skewness and ceiling effects could, of course, also depend on the respondents feeling quite well one month after surgery and therefore selecting the “none” response alternative more frequently, although, this is not the most likely explanation, as colorectal cancer surgery is a major procedure. Despite ceiling effects at the item level, all response alternatives were used for all items. In addition, there was an acceptable rate of missing items, pointing to good data quality. One exception was the item measuring *sexual activity*. This item demonstrated a high proportion of missing answers. Questions concerning sexuality are known to be sensitive and sometimes experienced as intrusive. Consequently, respondents might refrain from answering [37]. Earlier research has acknowledged sexuality as a problem area after colorectal surgery that is often disregarded by healthcare [38, 39]. Therefore, questions about sexuality and sexual function should be asked in a proper way to encourage respondents to answer. This underpins the importance of checking the wording of items during an instrument's development, for example, by conducting cognitive interviews.

The PRP was developed as a multidimensional instrument that includes five dimensions. However, it is not described anywhere how the division was made, and in this study, item-total correlations showed signs of unidimensionality. The following CFA confirmed problems with discriminating dimensions as the proposed dimensions were strongly correlated with each other. This indicates that the dimensions cannot be calculated separately because they likely do not reflect different aspects of recovery. In addition, most items had low correlations with their designated dimensions, which means that they are weak indicators. The PRP is intended to measure aspects of postoperative recovery. However, theoretical conclusions that can be drawn from such models are dependent on the direction of the causality between items and dimensions, and misspecification can lead to Type I or Type II errors [40]. Because the multidimensionality of the PRP is associated with great uncertainty, recovery should not be calculated at a dimensional level when using the current version of the PRP.

### 4.1. Methodological Limitations

A potential methodological limitation is the relatively low sample size (*n* = 154). According to the COSMIN study design checklist, a sample of at least 100 persons would be sufficient to produce methodologically sound estimates [41]. Regarding the CFA, it has been argued that model fit measures are sensitive to sample size. For example, chi-square statistics performed in large samples tend to reject the models, whereas small samples result in lack of statistical power. However, the measures for model fit that were reported in this study function well with small samples [33].

## 5. Conclusions

The PRP is an instrument that has shown promising properties during development and initial testing. However, the results of this study indicate that the instrument needs to be further developed and undergo a thorough psychometric evaluation before it can be used as a reliable and valid tool. As a suggestion, future studies should test measurement functioning in more depth, preferably using modern test theory. Furthermore, future studies should also focus on a revision of the scoring procedure at a dimensional level. However, discriminant validity issues need to be solved first, and meanwhile, recovery should not be calculated or reported at a dimensional level.

## Figures and Tables

**Table 1:** Correspondence between number of items responded by “none” and level of recovery constituting the original scoring procedure as recommended by Allvin et al. [28].

Number of items	Level of recovery
19	Fully recovered
15–18	Almost fully recovered
8–14	Partly recovered
7	Slightly recovered
0–6	Not at all recovered

**Table 2:** Model fit measures with recommended thresholds.

Measure	Results	Thresholds^†^
CMIN/DF	1.898^*∗∗∗*^	<3 good
CFI	0.895	>0.95 great, >0.9 acceptable, and >0.8 sometimes acceptable
GFI	0.810	>0.95
AGFI	0.746	>0.8
SRMR	0.0672	<0.09
RMSEA	0.086^*∗∗∗*^	<0.05 good, 0.05-0.1 moderate, and >0.1 bad

^†^Thresholds as recommended by Hu and Bentler [34]. ^*∗∗∗*^*p* value <0.001.

**Table 3:** Patients' characteristics.

Characteristics	
Gender (*n*, %)	
Male	79 (51.3)
Female	75 (48.7)

Age (years)	
Mean (SD)	69.4 (10.9)

Length of stay (days)	
Mean (SD)	10.1 (4.9)

Duration of surgery (minutes)	
Mean (SD)	276.9 (119.7)

Type of surgery (*n*, %)	
Low anterior resection of rectum	59 (38.3)
Abdominoperineal rectal resection	31 (20.1)
Colectomy	2 (1.3)
Left-sided hemicolectomy	5 (3.2)
Right-sided hemicolectomy	34 (22.1)
Sigmoid resection	23 (14.9)

PRP total score^†^	
Mean (SD)	61 (10.5)
Md	64
Min-max	27–76

PRP: the postoperative recovery profile, ^†^total score was used as a scoring procedure in the current study.

**Table 4:** Descriptive statistics for the items within the PRP instrument.

Item	Frequency distribution of response categories^†^, %				Missing, *n*(%)	Valid, *n*(%)	Item mean	Item SD	Md	Skewness	Item-totalcorrelation
	1	2	3	4							
^#^1. pain	2.0	13.8	45.4	38.8	2 (1.3)	152 (98.7)	3.21	0.751	3	−0.653	0.515
^#^2. Nausea	0.6	5.2	15.6	77.9	1 (0.6)	153 (99.4)	3.72	0.590	4	−2.174	0.509
^#^3. Gastrointestinal function	4.5	13.6	33.1	46.8	3 (1.9)	151 (98.1)	3.25	0.864	3	−0.938	0.355
^#^4. Fatigue	3.2	32.5	45.5	17.5	2 (1.3)	152 (98.7)	2.78	0.771	3	−0.043	0.655
^#^5. Muscle weakness	3.9	22.1	48.1	26.0	0 (0)	154 (100)	2.96	0.799	3	−0.396	0.655
^#^6. Appetite change	5.8	13.0	34.4	46.8	0 (0)	154 (100)	3.22	0.887	3	−0.961	0.631
^#^7. Sleeping difficulties	7.1	22.1	35.1	35.7	0 (0)	154 (100)	2.99	0.932	3	−0.526	0.598
^#^8. Anxiety and worry	7.1	16.9	30.5	44.8	1 (0.6)	153 (99.4)	3.14	0.946	3	−0.798	0.727
^#^9. Feeling down	7.8	13.0	31.2	46.8	2 (1.3)	152 (98.7)	3.18	0.945	3	−0.950	0.769
^#^10. Reestablishing everyday life	11.0	22.7	37.7	27.9	1 (0.6)	153 (99.4)	2.83	0.965	3	−0.408	0.709
^#^11. Sexual activity	22.1	9.7	14.9	37.7	24 (15.6)	130 (84.4)	2.81	1.258	3	−0.436	0.530
^#^12. Social activities	5.2	14.9	31.2	46.8	3 (1.9)	151 (98.1)	3.22	0.894	3	−0.900	0.725
^#^13. Personal hygiene	5.2	20.8	73.4	99.4	1 (0.6)	153 (99.4)	3.69	0.567	4	−1.650	0.646
^#^14. Interest in surroundings	1.3	3.9	9.7	83.8	2 (1.3)	152 (98.7)	3.78	0.574	4	−2.937	0.536
^#^15. Bladder function	3.9	9.1	20.8	64.9	2 (1.3)	152 (98.7)	3.49	0.822	4	−1.554	0.442
^#^16. Mobilization	2.6	13.0	35.7	48.1	1 (0.6)	153 (99.4)	3.30	0.795	3	−0.913	0.729
^#^17. Feeling lonely/abandoned	2.6	8.4	18.8	70.1	0 (0)	154 (100)	3.56	0.758	4	−1.730	0.713
^#^18. Dependence on others	1.3	11.7	23.5	54.5	0 (0)	154 (100)	3.40	0.746	4	−1.003	0.674
^#^19. Difficulties in concentration	0.6	8.4	32.5	57.8	1 (0.6)	153 (99.4)	3.48	0.680	4	−1.087	0.666

*n* = number; SD = standard deviation; md = median; ^†^response categories were 1 = severe, 2 = moderate, 3 = mild, and 4 = none.

## Data Availability

The data that support the findings of this study are available from the corresponding author upon reasonable request.
